# Short- and Long-Term Self-Reported Symptoms in Adolescents Aged 12–19 Years after Vaccination against SARS-CoV-2 Compared to Adolescents Not Vaccinated—A Danish Retrospective Cohort Study

**DOI:** 10.3390/vaccines10111863

**Published:** 2022-11-04

**Authors:** Selina Kikkenborg Berg, Helle Wallach-Kildemoes, Line Ryberg Rasmussen, Ulrikka Nygaard, Henning Bundgaard, Maria Nivi Schmidt Petersen, Cecilie Bech Hammer, Annette Kjær Ersbøll, Lau Caspar Thygesen, Susanne Dam Nielsen, Anne Vinggaard Christensen

**Affiliations:** 1 Department of Cardiology, Rigshospitalet, Copenhagen University Hospital, Inge Lehmanns Vej 7, 2100 Copenhagen, Denmark; 2 Faculty of Health and Medical Sciences, University of Copenhagen, Blegdamsvej 3B, 2200 Copenhagen, Denmark; 3 Department of Paediatrics and Adolescents Medicine, Rigshospitalet, Copenhagen University Hospital, Blegdamsvej 9, 2100 Copenhagen, Denmark; 4 National Institute of Public Health, University of Southern Denmark, Studiestræde 6, 1455 Copenhagen, Denmark; 5 Department of Infectious Disease, Rigshospitalet, Copenhagen University Hospital, Blegdamsvej 9, 2100 Copenhagen, Denmark

**Keywords:** COVID-19, vaccine, BNT162b2, adolescents, side effects

## Abstract

This study investigated self-reported short- and long-term symptoms among adolescents receiving the BNT162b2 (Pfizer/BioNTech) vaccine against SARS-CoV-2 and those who did not. A retrospective cohort study based on Danish national survey (collected between 20 July and 15 September 2021) and register data was conducted. Differences in short-term (<14 days) and long-term (>two months) symptoms were explored using logistic regression adjusted for confounders. A total of 747 vaccinated (first dose n = 326; second dose n = 421) and 6300 unvaccinated adolescents were included in analyses of short-term symptoms and 32 vaccinated and 704 unvaccinated adolescents in long-term symptom analyses. In the first 14 days after the first and second vaccine dose the most reported symptoms included headache and muscle or joint symptoms. In both vaccinated and unvaccinated adolescents, the 15–19-year-olds reported significantly higher proportions of all symptoms compared to the 12–14-year-olds. After the second vaccine dose vaccinated 12–14-year-olds reported significantly more headache in adjusted analyses (OR 2.20 (95% CI 1.24; 3.90)). Among the 15–19-year-olds, significantly more vaccinated adolescents reported gastrointestinal symptoms (1.38 (1.06; 1.81)), headache (1.66 (1.24; 2.22)), and tiredness (1.44 (1.08; 1.93)). No differences were found in long-term symptoms. Vaccinated adolescents reported significantly more short-term symptoms including headache, tiredness, and gastrointestinal symptoms after the second vaccine dose than unvaccinated adolescents. Long-term symptom results should be interpreted with caution due to limited sample size.

## 1. Introduction

On 10 May 2021 the Food and Drug Administration approved the first COVID-19 vaccine BNT162b2 (Pfizer-BioNTech) for emergency use in adolescents aged 12–15 years based on data from a study by Frenck et al. [1]. The RCT included 2260 adolescents aged 12–15 years who received two vaccine doses. Results showed that there were more local and systemic symptoms seven days after each vaccine dose in the group receiving the BNT162b2 vaccine than in the group receiving a placebo and that more symptoms were reported after the second vaccine dose. The most common local symptom was pain at the injection site (79% vs. 18% after the second vaccine dose). The most common systemic symptoms were fatigue (66% vs. 25%), headache (65% vs. 24%), chills (42% vs. 7%), and muscle pain (32% vs. 8%). Furthermore, systemic symptoms explored were fever (20% vs. 1%), vomiting (3% vs. 1%), diarrhea (6% vs. 4%), joint pain (16% vs. 5%), and antipyretic use (51% vs. 9%). Both local and systemic symptoms were generally mild to moderate and typically resolved within 1–2 days. Any adverse events were reported by 6% in both the BNT162b2 and placebo groups in the period from receiving the first dose until one month after the second. Among BNT162b2 recipients, severe adverse events were reported by 0.6% [1]. Besides the study by Frenck et al., few others have described common self-reported acute side effects of the BNT162b2 vaccine. In accordance with Frenck et al., an American study combining Vaccine Adverse Event Reporting System (VAERS) and survey data found the most common reported symptoms among children up to 15 years to be fatigue, chills, pyrexia, pain, and headache after the BNT162b2 vaccine [2]. Similarly, a systematic review and meta-analysis of effectiveness and safety of SARS-CoV-2 vaccines among children and adolescents showed the most common symptoms after the second vaccine dose to be fatigue, injection-site pain, headache, chills, and myalgia/muscle pain were the top five adverse events after the second dose of SARS-CoV-2 vaccines [3]. Cases of myocarditis and pericarditis [4,5,6,7] and Guillain-Barré syndrome [8,9] have also been associated with the BNT162b2 vaccine, mostly presented in case series/reports.

In Denmark, the BNT162b2 COVID-19 vaccine was recommended from May 2021 and July 2021 for adolescents aged 16–19 years and 12–15 years, respectively. As of July 2022, 78.4% of adolescents aged 12–15 years and 88.2% aged 16–19 years had received the vaccine [10].

We aimed to investigate self-reported short- and long-lasting symptoms in adolescents following BNT162b2 vaccination in this study using real-life data from the large national LongCOVIDKidsDK [11,12] survey. 

### Objective

To investigate self-reported short-term symptoms among 12–19-year-olds and long-term symptoms among 15–19-year-old adolescents who received the BNT162b2 vaccine against SARS-CoV-2 and those who did not. 

## 2. Materials and Methods

### 2.1. Study Design

The present Danish retrospective cohort study combines data from the LongCOVIDKidsDK [11,12] survey with follow-up using national registers. Conducted between 20 July and 15 September 2021, the survey was designed to explore health and symptoms in SARS-CoV-2 positive adolescents and a 1:4 matched (sex and age) reference group without a positive SARS-CoV-2 test. The LongCOVIDKidsDK survey included a long range of symptoms consistent with those often reported after receiving the BNT162b2 vaccine.

### 2.2. Data Sources

The LongCOVIDKidsDK survey was sent by mail via a secure digital post-box and answered in a web application for online surveys (REDCap) from 20 July to be answered by 15 September 2021 at the latest, with one initial invitation and two reminders. For participants aged 12–14 years, the symptoms were reported by a parent for the adolescent (proxy for self-reported symptoms). Symptoms among 15–19-year-olds were self-reported.

Survey data were obtained using the Children’s Somatic Symptoms Inventory (CSSI-24) [13] validated questionnaire as well as ancillary questions regarding the 23 most common symptoms of long COVID identified from the Long COVID Kids Rapid Survey, January 2021 [14]. The CSSI-24 is a 24-item generic questionnaire identifying the presence of various somatic symptoms in children. The items are scored on a five-point Likert scale from zero (not at all) to four (a whole lot) in the past two weeks (short-term symptoms). For the 23 questions regarding the long COVID symptoms, participants were asked to rate the intensity (as for the short-term symptoms) and the duration of the symptom with a recall period of three months. Furthermore, questions were included about height and weight, calculating weight status according to the WHO classification of BMI in children and adolescents [15].

National register data were accessed through The Danish Health Data Authority. Individual-level data from the Danish LongCOVIDKidsDK survey [11,12] were linked to the Danish national COVID register [16] to obtain information on COVID-19 vaccination and SARS-CoV-2 test results to form the study populations. To form demographic and clinical profiles for the included adolescents, the following information was obtained from the Danish registers: hospital diagnoses (the Danish National Patient Register [17]), filled prescription drugs (Danish Prescription Registry [18]), parental highest attained education [19], family income [20] and the Danish Civil Registration System with information on Danish residency and family identification. 

### 2.3. Setting

The LongCOVIDKidsDK survey data were obtained in a period when Denmark was not in lockdown. 

### 2.4. Study Population and Study Groups

Using the Danish national registers, adolescents from the LongCOVIDKidsDK survey were retrospectively divided into groups according to vaccine exposure: participants vaccinated against COVID-19 prior to survey completion and those not. 

Among those returning the questionnaire the following participants were excluded: (1) adolescents with a positive SARS-CoV-2 test before the date of survey completion, (2) adolescents receiving a COVID-19 vaccine other than BNT162b2 (Pfizer-BioNTech), (3) adolescents with more than 42 days between first and second vaccination dose, and (4) adolescents who were not residents in Denmark at least one year before survey completion.

Furthermore, specific time spans between vaccination and survey completion were required in vaccinated adolescents to cover either the short- or long-term symptoms. For short-term symptoms, only participants with survey completion exactly 14 days after the first or second vaccine dose were included. For long-term symptoms, participants with survey completion within 12–16 weeks after their second vaccine and with a recall period of three months were included. For long-term symptoms, unvaccinated participants also had a recall period of three months; apart from that, no such restrictions were applied to the unvaccinated population. Due to the three months follow-up after the second vaccine dose, it was only possible to include adolescents aged 15–19 in the analyses for the long-term symptoms. The index date for each participant was the date of survey completion.

The following study groups were applied based on the above definitions: 

#### 2.4.1. Study Groups for Short-Term Symptoms

Exposed study groups:

1. Adolescents receiving the first vaccine dose exactly 14 days before survey completion.
2. Adolescents receiving the second vaccine dose exactly 14 days before survey completion.

Unexposed study group: Adolescents not vaccinated at the time of survey completion. 

#### 2.4.2. Study Groups for Long-Term Symptoms

Exposed study group: Adolescents with three months recall in the survey receiving the second vaccine dose within 12–16 weeks before survey completion.

Unexposed study group: Adolescents with three months recall in the survey not vaccinated at the time of survey completion. 

**Figure 1:**
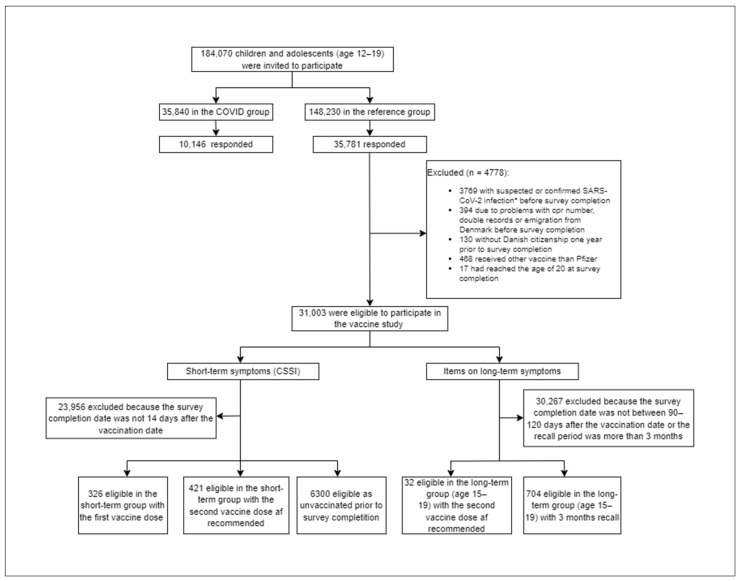
Flowchart. * Adolescent in the reference group who reported that they suspected having been infected with SARS-CoV-2 but did not have access to a test at the time were considered to have suspected SARS-CoV-2 infection.

A total of 180,000 adolescents were invited to participate in the survey, and 35,781 responded (25.2%). Of these, 747 vaccinated adolescents (326 as the first dose population and 421 as the first as well as the second dose population) and 6300 unvaccinated adolescents were eligible to be included in analyses of short-term symptoms (Figure 1). In the analyses of long-term symptoms, 32 vaccinated adolescents and 704 unvaccinated adolescents were included (Figure 1). 

### 2.5. Outcome Measures

#### 2.5.1. Dichotomization

For both short- and long-term symptoms all responses were dichotomized into yes (almost never, sometimes, often, almost always) or no (never).

#### 2.5.2. Grouping of Symptoms

In total, 20 of the 24 short-term symptoms from CSSI-24 were grouped into seven symptom categories (number of individual symptoms in parenthesis): central nervous system (CNS) symptoms (5), cardiopulmonary symptoms (3), gastrointestinal symptoms (4), muscle or joint symptoms (4) headache (1), chills (1), and tiredness (2) (Supplementary Table S1).

The long-term symptoms were defined as symptoms lasting at least two months. In total, 14 of the 23 long-COVID symptoms were included and grouped into ten groups: headache, trouble remembering or concentrating, neurological symptoms (2), cardiopulmonary symptoms (3), gastrointestinal symptoms (2), pain in muscles/joints, fatigue, rashes, fever, mood swings (Supplementary Table S1).

For grouped symptoms, a participant was considered as having the symptom group of interest if they had a yes in at least one of the dichotomized individual symptoms. 

### 2.6. Risk Factors for Outcome

Several diseases and health conditions can be considered to be risk factors for the symptoms included as outcomes in the present analyses. Therefore, groups of diseases and prescription drugs that are possible risk factors for the outcomes were identified for each of the seven symptom groups. Identification was done from primary discharge diagnosis (ICD-10 codes [21]) in the Danish National Patient Register [17] and prescription drug proxies, e.g., insulins (ATC codes [22]) in the Danish National Prescription Register [18]. Different look back periods (time prior to the index date) were utilized as diseases may be non-persistent (i.e., infectious diseases and injuries) or persistent (Supplementary Table S2).

Participants with the identified diseases corresponding to risk factors for outcome (Supplementary Table S2) were excluded from analyses of the outcome of interest, e.g., participants with migraine were excluded from analyses with headache as an outcome.

### 2.7. Prevalent Somatic and Psychiatric Disorders Associated with Exposure and Outcome

Disorders prior to the index date were identified by applying a list of somatic disorders and health conditions in adolescents conferring an increased risk of SARS-CoV-2 infection assessed by the Danish Pediatric Society (DPS) [23]. By means of an analogous approach to risk-factor assessment, individuals with this list of disorders prior to the index date were identified based on primary discharge diagnoses and/or prescription drug use proxies. Assuming the DPS list of prevalent disorders is also associated with being vaccinated against COVID-19, the prevalent somatic disorders were applied in the statistical analyses as a dichotomous confounder variable for the somatic disorders (yes, if at least one somatic disorder registered, and no, if none) (Supplementary Tables S3 and S4).

### 2.8. Statistical Analyses

Initially, a descriptive analysis of baseline characteristics of adolescents at index date was performed including age, sex, body mass index (BMI), risk factors for outcome, prior psychiatric disease (any psychiatric ICD-10 diagnosis) and certain somatic disorders, recent prescription filling on selected drugs, along with parental socioeconomic position (highest attained education and household income). Educational level was grouped into basic education, high school or vocational training, and higher education. The highest educational level in the household was applied. The latest available household income (tax and member-adjusted household income) was from 2019 and was divided into tertiles. BMI was calculated based on self-reported weight and height. Due to some very high reported weights, BMI ≥ 41 was recoded as missing. 

In addition, a descriptive analysis was conducted comparing non-responders with responders. For the non-responders, the age-group median survey completion date among responders was applied as the index date.

For results with less than five individuals per cell, numbers are presented as <5 and percentages are masked due to data protection rules from the Danish Health Data Authority. 

#### Uni- and Multivariable Regression Analyses

Uni- and multivariable logistic regression models were used to estimate odds ratios (OR) with 95% confidence intervals (CI) for each symptom group in vaccinated versus unvaccinated adolescents for both short- and long-term symptoms. For each short-term symptom group, the identified risk factors were accounted for by excluding participants with risk factors for the symptom outcome (Supplementary Table S2). As symptom reporting most likely differs according to parent reporting on the child’s symptoms or self-reported symptoms, all analyses were stratified by age group. Multivariable logistic regression models were performed, applying two sets of confounders: (1) prevalent somatic disorder (yes/no) and psychiatric disorder, and (2) obesity and household income (as these variables have missing information) along with survey-completion time period for participants aged 15–19 years. 

No correction for multiple testing was done. Comparison of symptoms was done on separate populations after first (seven comparisons) and second (seven comparisons) vaccine dose and for long-term symptoms (10 comparisons). 

The significance level was set at <0.05. All analyses were conducted using StataCorp. 2021. Stata Statistical Software: release 17. 

### 2.9. Patient and Public Involvement

Due to the urgency of the study question, there was no patient or public involvement in defining the research question, study design, outcome measures or the conduct of the study.

## 3. Results

### 3.1. Participants

Characteristics of vaccinated and unvaccinated adolescents for short- and long-term symptoms are presented in Table 1. There was a higher proportion of risk factors and prevalent diseases among vaccinated adolescents, e.g., muscle and joint conditions and respiratory conditions. Furthermore, parents of adolescents in the vaccinated groups had higher educational levels and higher incomes than parents of unvaccinated adolescents. 

### 3.2. Outcomes

#### 3.2.1. Short-Term Symptoms

After the first vaccine dose the most commonly reported symptoms among 12–14-year-olds were headache (unvaccinated 41.2%; vaccinated 40.5%), muscle or joint symptoms (42.7%; 38.9%) and gastrointestinal symptoms (39.0; 33.0). Few 12–14-year-olds reported cardiopulmonary symptoms (11.0; 9.3%). Among the 15–19-year-olds the most common symptoms were tiredness (unvaccinated 69.0%; vaccinated 66.1%), headache (64.9%;65.0%), and muscle or joint symptoms (65.7%; 58.9%). The least reported symptoms in the age group were cardiopulmonary symptoms (44.2; 40.0%) and chills (44.2%; 45.5%). For all symptoms after the first vaccine dose, the 15–19-year-olds reported significantly higher proportions compared to the 12–14-year-olds. This applied for both vaccinated and unvaccinated adolescents (Table 2). However, the difference in proportions of symptoms between the vaccinated and unvaccinated adolescents was not significantly different between the 12–14-year-olds and the 15–19-year-olds (Supplementary Table S5).

There were no differences in reporting of symptoms between vaccinated and unvaccinated adolescents in the multivariate adjusted analyses (Table 3). 

After the second vaccine dose the most common symptoms among adolescents aged 12–14-years were headache (unvaccinated 41.2%; vaccinated 61.5%), muscle or joint symptoms (42.7%; 45.7%) and gastrointestinal symptoms (39.0; 44.2). Few 12–14-year-olds reported cardiopulmonary symptoms (11.0; 9.6%). Among the 15–19-year-olds the most common symptoms were tiredness (unvaccinated 69.0%; vaccinated 78.1%), headache (64.9%; 77.4%), and muscle or joint symptoms (65.7%; 62.5%). The least reported symptoms in the age group were cardiopulmonary symptoms (44.2; 42.9%) and chills (44.2%; 46.7%)

Among both vaccinated and unvaccinated adolescents, the 15–19-year-olds reported significantly higher proportions of all symptoms compared to the 12–14-year-olds (Table 2). The difference in proportions of symptoms between the vaccinated and unvaccinated adolescents was, however, not significantly different between the 12–14-year-olds and the 15–19-year-olds (Supplementary Table S5).

The multivariate analysis showed that the vaccinated adolescents in both age groups during the first 14 days after the second dose reported more headache (12–14 years old: OR 2.20 (95% CI 1.24 to 3.90), *p* = 0.007, 15–19 years old: 1.66 (1.24 to 2.22), *p* = 0.001) compared to unvaccinated adolescents in adjusted analyses. In the age group 15–19 years, the vaccinated group further reported more gastrointestinal symptoms (1.38 (1.06 to 1.81), *p* = 0.018) and tiredness (1.44 (1.08 to 1.93), *p* = 0.014) compared to the unvaccinated group (Table 4). 

#### 3.2.2. Long-Term Symptoms

The most common long-term symptoms reported by 15–19-year-olds were fatigue (vaccinated 46.9%; unvaccinated 23.2%), mood swings (34.4; 21.3) and trouble remembering or concentrating (28.1%; 19.3%). Few reported fever (n < 5; n < 5), rashes (n < 5; 5.4%), and pain in muscles/joints (n < 5; 5.7%). After adjustment, no differences were seen in reporting of long-term symptoms between the two groups (Table 5). 

#### 3.2.3. Non-Responders

There were more girls among responders than non-responders. Among the 12–14-year-olds there were more unvaccinated among the responders (58.8%) than the non-responders (46.2%). For the 15–19-year-olds, it was the opposite with 53.8% unvaccinated among non-responders and 41.2% among the responders. Moreover, there was a tendency among responders in both the vaccinated and unvaccinated groups to have parents with higher income and educational levels than the non-responders (Supplementary Table S6). 

## 4. Discussion

In this study, self-reported symptoms were explored among 12–19-year-old Danish adolescents who had received the BNT162b2 vaccine against COVID-19 and unvaccinated adolescents. For the short-term symptoms, no difference in symptoms was found between groups after the first vaccine dose. Vaccinated 12–19-year-olds reported significantly more headache within the first 14 days after the second vaccine dose compared to the unvaccinated group. Furthermore, vaccinated 15–19-year-olds also reported significantly more gastrointestinal symptoms and tiredness. There was no difference between groups in the long-term symptoms.

### 4.1. Strengths and Limitations

The LongCOVIDKidsDK study is based on real-life data and was carried out just as the vaccinations programme for adolescents started, which strengthens this study. This was also before the emergence of the Omicron variant of SARS-CoV-2 which has since infected almost all Danish children and adolescents. This is therefore a unique cohort as this study is impossible to repeat. 

Another strength is the access to the Danish registers with nationwide coverage, including the Danish vaccination register, which allowed for the identification of adolescents who had received the BNT162b2 vaccine. Furthermore, we did extensive work to account for existing morbidity among adolescents for each symptom group. Adolescents with pre-existing conditions that could result in a reporting of that symptom (risk-factors for outcome) were excluded to avoid bias in analyzing symptom-outcome comparing vaccinated with unvaccinated. 

Where the adolescents aged 15–19 years answered the survey themselves, the survey for adolescents aged 12–14 years was parent-proxy-reported. Parental reporting, however, is a valid proxy for child self-report of health-related quality of life [24]. There may have been possible seasonal variations in the period of data collection (20 July–15 September 2021) in terms of a respiratory syncytial virus epidemic that peaked by the end of August 2021. Therefore, analyses were adjusted for the survey completion time period. Furthermore, there was markedly more illness among adolescents during the summer and fall of 2021, likely due to the lockdown in the previous year. 

Besides differences in symptoms reported between vaccinated and unvaccinated adolescents, this study also explores the frequency of various symptoms among both vaccinated and unvaccinated as well as differences in symptoms reported between 12–14-year-olds and 15–19-year-olds.

The study also has limitations. Selection bias might be present in response/non-response and vaccinated/not vaccinated. Overall, vaccinated adolescents tended to have higher prevalence of risk factors and co-morbidities compared to the unvaccinated adolescents, and those aged 15–19 reported more symptoms for each symptom group compared to the 12–14-years-old. There is a risk that adolescents themselves or the parents of adolescents with more severe symptom burden could be more prone to respond to the survey. 

In the analyses, symptoms responses were grouped as no (never) or yes (almost never, sometimes, often, almost always). Thus, the intensity of symptoms is not explored in the present paper. This could, however, be relevant for further study in future research. 

The small sample size in the analyses of long-term symptoms is a limitation and the results should therefore be interpreted with caution. Moreover, in logistic regression model intercept correction is recommended for rare event data [25]. However, to our knowledge this has not been reported before and therefore we believe that the results are of interest and thus, they are presented in the present study. 

### 4.2. Comparisons with Other Studies

The present study shows that among the 12–14-year-olds, the vaccinated adolescents reported more headache than the unvaccinated after the second vaccine dose, whereas the vaccinated 15–19-year-olds reported more headache, tiredness, and gastrointestinal symptoms, which is expected after being vaccinated [1,3,26,27]. After the first vaccine dose, no difference was found in self-reporting of symptoms between the vaccinated and unvaccinated. The study by Frenck et al. found that the most common symptoms among 12–15-year-olds after the first dose were fatigue (60.0% vs. 41.0%), headache (55.0% vs. 35.0%), and chills (28.0% vs. 10.0%). After the second vaccine dose the vaccinated adolescents aged 12–14 reported more headache than the unvaccinated ones (61.5% vs. 41.2%). Among adolescents aged 12–15, Frenck et al. found that 65% of vaccinated adolescents reported headache compared to 24% in the placebo group. For the vaccinated adolescents aged 15–19, we found that they reported more headache (77.4% vs. 64.9%), tiredness (78.1% vs. 69.0%) and gastrointestinal symptoms (66.9% vs. 59.9%) than the unvaccinated ones. This is in line with the findings of Frenck et al., who found 61.0% vs. 24% reporting headache, 66% vs. 23% reporting fatigue, and 3%/8% vs. 2%/5% reporting vomiting and diarrhea, respectively. The participants in our study reported more gastrointestinal symptoms compared to the Frenck et al. study. An explanation for this may be differences in gastrointestinal symptoms listed in the two studies. The present study reported on nausea or upset stomach, vomiting, loose (runny) bowel movements or diarrhea and pain in the stomach or abdomen, whereas Frenck et al. report on vomiting and diarrhea only. A study from Saudi Arabia presents self-reported symptoms from 965 adolescents aged 12–18 years following the BNT162b2 vaccine [26]. The most common reported side effects were pain or redness at the site of injection (90%), fatigue (67%), fever (59%), headache (55%), nausea or vomiting (21%), and chest pain and shortness of breath (20%) among adolescents receiving one or two doses of the vaccine. More side effects were seen after the second vaccine dose. The frequency of gastrointestinal symptoms was also higher compared to the present study. Furthermore, the proportion reporting cardiopulmonary symptoms was higher than in the 12–14-year-olds in the present study but lower than in its 15–19-year-olds. The differences could be due to the definition of symptoms. The study did not include a control group [26]. In an American study by Flora et al. some of the most common survey-reported symptoms were fatigue (63.4%) and headache (35.8%) among BNT162b2 recipients [2]. This is consistent with the 15–19-year-olds in the present study reporting tiredness (78.1%) and headache (77.1%) as the most common symptoms after the second vaccine dose. Among the 12–14-year-olds the most common symptom was headache (61.55%). This age group, however, reported significantly less tiredness than the 15–19-year-olds (51.9%). Other frequent symptoms in the American study were chills (43.9%) and pyrexia (43.1%). In the present study, chills were reported by 30.8% of 12–14-year-olds and 46.7% of 15–19-year-olds. The younger adolescents generally report lower proportions of symptoms and as the age groups are pooled in the American study this might explain the relatively lower proportions of symptoms reported in the American study compared to the present study [2].

Another study, from Thailand, explored cardiovascular effects after the second dose of the BNT162b2 vaccine in 301 adolescents aged 13–18 years [28]. The most common self-reported cardiovascular effects were tachycardia (7.6%), shortness of breath (6.6%), palpitations (4.3%), chest pain (4.3%), and hypertension (3.9%). In the present study those symptoms were grouped together making direct comparisons difficult [28].

In the present study, the unvaccinated group generally reported high rates of symptoms. An explanation for this could be the large burden of illness among adolescents in general in the summer of 2021, including the respiratory syncytial virus epidemic. 

For long-term symptoms, no differences were found between groups; however, results should be interpreted with caution due to the limited sample size. It is known that some adolescents experienced severe adverse events after receiving the vaccination against COVID-19, such as myocarditis and multisystem inflammatory syndrome [5,6,29,30,31]. This is, however, very rare and not captured in the present analyses. Severe adverse events were outside the scope of this study.

Future studies should investigate the ICD-10 diagnostic profile of adolescents before and after vaccination against COVID-19 to look for differences between vaccinated and unvaccinated adolescents that extend the self-reported symptom profile and with longer follow-up. These will be reported from the LongCOVIDKidsDK study. 

## 5. Conclusions 

This retrospective cohort study compares self-reported symptoms among 12–19-year-old Danish adolescents vaccinated against COVID-19 with the BNT162b2 vaccine with unvaccinated adolescents. More vaccinated adolescents reported headache during the first 14 days after the second vaccine dose than unvaccinated adolescents. For the 15–19-year-olds only, vaccinated adolescents reported more gastrointestinal symptoms and tiredness than unvaccinated ones. No differences were found in long-term symptoms. The results should, however, be interpreted with caution due to the limited sample size. 

## Figures and Tables

**Table 1:** Characteristics of vaccinated and unvaccinated adolescents with survey completion on short-term and long-term symptoms.

	Short-Term Symptoms ^1^			Long-Term Symptoms ^2^	
	Vaccinated First Dose	Vaccinated Second Dose	Unvaccinated	Vaccinated Second Dose	Unvaccinated
	(n = 326)	(n = 421)	(n = 6300)	(n = 32)	(n = 704)
Age (mean, SD)	14.6 (1.5)	17.1 (1.6)	14.8 (1.9)	18.0 (1.0)	16.6 (1.4)
Age group 12–14 years, n (%)	205 (62.9%)	52 (12.4%)	3702 (58.8%)	-	-
Age group 15–19 years, n (%)	121 (37.1%)	369 (87.6%)	2598 (41.2%)	32 (100.0%)	704 (100.0%)
Sex (Girl), n (%)	167 (51.2%)	251 (59.6%)	3210 (51.0%)	25 (78.1%)	379 (53.8%)
BMI (mean, SD) ^3^	20.1 (3.1)	21.7 (3.7)	20.0 (3.6)	22.1 (4.5)	21.5 (3.7)
Obesity, n (%) ^3^	7 (2.1%)	21 (5.0%)	216 (3.4%)	(n < 5)	33 (4.7%)
**Risk factors for outcome, n (%) ^4^**					
Muscle and joint conditions	44 (13.5%)	71 (16.9%)	862 (13.7%)	8 (25.0%)	111 (15.8%)
Cardiovascular conditions	(n < 5)	(n < 5)	17 (0.3%)	(n < 5)	(n < 5)
CNS conditions	5 (1.5%)	10 (2.4%)	110 (1.7%)	5 (15.6%)	16 (2.3%)
Headache	(n < 5)	6 (1.4%)	45 (0.7%)	(n < 5)	6 (0.9%)
Gastrointestinal conditions	7 (2.1%)	7 (1.7%)	100 (1.6%)	10 (31.3%)	13 (1.8%)
Respiratory conditions	24 (7.4%)	31 (7.4%)	333 (5.3%)	(n < 5)	26 (3.7%)
Febrile conditions	(n < 5)	(n < 5)	97 (1.5%)	(n < 5)	13 (1.8%)
**Prevalent disorders as confounders, n (%) ^5^**					
Asthma and other respiratory disorders	20 (6.1%)	24 (5.7%)	290 (4.6%)	(n < 5)	22 (3.1%)
Cardiovascular or renal disorders	(n < 5)	(n < 5)	22 (0.3%)	(n < 5)	(n < 5)
Gastrointestinal disorders	(n < 5)	(n < 5)	43 (0.7%)	7 (21.9%)	7 (1.0%)
Endocrine disorders incl. diabetes	(n < 5)	7 (1.7%)	33 (0.5%)	(n < 5)	6 (0.9%)
Hematological disorders	-	(n < 5)	39 (0.6%)	(n < 5)	(n < 5)
Rheumatological disorders	-	(n < 5)	23 (0.4%)	(n < 5)	(n < 5)
Neuromuscular disorders	(n < 5)	(n < 5)	11 (0.2%)	(n < 5)	-
Congenital malformations & chromosomal abnormalities	(n < 5)	5 (1.2%)	39 (0.6%)	(n < 5)	(n < 5)
Malignancy or organ transplantation	(n < 5)	(n < 5)	31 (0.5%)	6 (18.8%)	7 (1.0%)
Any of the above somatic disorders	49 (15.0%)	71 (16.9%)	763 (12.1%)	19 (59.4%)	81 (11.5%)
Psychiatric disorder	25 (7.7%)	37 (8.8%)	518 (8.2%)	11 (34.4%)	70 (9.9%)
**Prevalent prescription-drug use, n (%)**					
Bronchodilating agents, short-acting	(n < 5)	(n < 5)	39 (0.6%)	(n < 5)	(n < 5)
Long-acting bronchodilating agents	10 (3.1%)	9 (2.1%)	136 (2.2%)	-	10 (1.4%)
Systemic antibiotics	(n < 5)	(n < 5)	35 (0.6%)	(n < 5)	8 (1.1%)
Analgesics: Paracetamol	(n < 5)	12 (2.9%)	86 (1.4%)	(n < 5)	15 (2.1%)
Analgesics: NSAIDs	7 (2.1%)	13 (3.1%)	125 (2.0%)	(n < 5)	23 (3.3%)
**Parental socio-economic position, n (%)**					
Highest attained parental education					
Basic education	9 (2.8%)	11 (2.6%)	296 (4.7%)	(n < 5)	52 (7.4%)
High school and vocational training	112 (34.4%)	182 (43.2%)	2382 (37.8%)	12 (37.5%)	293 (41.6%)
Higher education	204 (62.6%)	227 (53.9%)	3598 (57.1%)	19 (59.4%)	355 (50.4%)
Annual family income					
Low (1. tertile)	71 (21.8%)	95 (22.6%)	2047 (32.5%)	6 (18.8%)	293 (41.6%)
Middle (2. tertile)	108 (33.1%)	127 (30.2%)	2025 (32.1%)	12 (37.5%)	203 (28.8%)
High (3. tertile)	145 (44.5%)	196 (46.6%)	2189 (34.7%)	14 (43.8%)	199 (28.3%)

^1^ Symptoms reported within the first 14 days after first and second vaccine dose. Among unvaccinated: Reported symptoms within last 14 days before survey completion. ^2^ Symptoms reported within 12–16 weeks after second dose vaccine. Among unvaccinated: Reported symptoms within 12–16 weeks before survey completion. ^3^ Median body mass index (BMI) is based on standard BMI calculation (kg/m^2^). Obesity is based on WHO BMI categories in children and adolescents. ^4^ Risk-factors for outcome (i.e., reported symptoms) correspond to registered prior hospital diagnoses and medicine proxies implying groups of reported symptoms (as defined in Supplementary Table S2). ^5^ Prevalent disorders included as confounders in the analyses: The included somatic disorders are included as one somatic disorder binary variable (1 if any, 0 if none). Psychiatric disorder if any registered F- ICD code.

**Table 2:** Proportion reporting categories of groups of symptoms during the last 14 days before survey completion: Participants vaccinated 14 days before survey completion versus unvaccinated.

Group of Survey Symptoms	Age-Group (Years)		14 Days after 1. Vaccine							14 Days after 2. Vaccine					
		Total	Unvaccinated			Vaccinated			Total	Unvaccinated			Vaccinated		
			N	Symptom (%)	*p*	N	Symptom (%)	*p*		N	Symptom (%)	*p*	N	Symptom (%)	*p*
**CNS symptoms ^1^**	**12–14**	3855	3653	20.1	<0.00	202	19.8	<0.00	3703	3653	20.1	<0.00	50	20.0	<0.00
	**15–19**	2656	2537	55.9		119	49.6		2898	2537	55.9		361	59.0	
**Cardiopulmonary symptoms ^2^**	**12–14**	3898	3694	11.0	<0.00	204	9.3	<0.00	3746	3694	11.0	<0.00	52	9.6	<0.00
	**15–19**	2709	2589	44.2		120	40.0		2955	2589	44.2		366	42.9	
**Gastrointestinal symptoms ^3^**	**12–14**	3851	3651	39.0	<0.00	200	33.0	<0.00	3703	3651	39.0	<0.00	52	44.2	<0.00
	**15–19**	2668	2549	59.9		119	56.3		2911	2549	59.9		362	66.9	
**Muscle or joint symptoms ^4^**	**12–14**	3404	3229	42.7	<0.00	175	38.9	<0.00	3275	3229	42.7	<0.00	46	45.7	0.03
	**15–19**	2316	2209	65.7		107	58.9		2513	2209	65.7		304	62.5	
**Headache ^5^**	**12–14**	3892	3687	41.2	<0.00	205	40.5	<0.00	3739	3687	41.2	<0.00	52	61.5	0.01
	**15–19**	2688	2568	64.9		120	65.0		2931	2568	64.9		363	77.4	
**Chills ^6^**	**12–14**	3854	3653	28.7	<0.00	201	25.9	<0.00	3705	3653	28.7	<0.00	52	30.8	0.03
	**15–19**	2671	2550	44.2		121	45.5		2916	2550	44.2		366	46.7	
**Tiredness ^7^**	**12–14**	3907	3702	37.6	<0.00	205	37.1	<0.00	3754	3702	37.6	<0.00	52	51.9	<0.00
	**15–19**	2719	2598	69.0		121	66.1		2967	2598	69.0		369	78.1	

^1^ CNS: numbness or tingling in parts of your body, blurred vision, heavy feelings in your arms or legs, and faintness or dizziness. ^2^ Cardiopulmonary: palpitations, dyspnea, and chest pain. ^3^ Gastrointestinal: nausea or upset stomach, vomiting, diarrhea, and pain in your stomach or abdomen. ^4^ Muscle or joint symptoms: pains in your lower back, sore muscles, pain in your knees, elbows, or other joints. ^5^ Headache: headache. ^6^ Chills: hot or cold spells (suddenly feeling hot or cold for no reason). ^7^ Tiredness: weakness (feeling weak) in parts of your body, feeling low in energy or slowed down.

**Table 3:** Odds ratio (OR) of short-term symptoms (first 14 days) after first dose vaccine against COVID-19: Results of logistic regression analyses on first dose vaccine population ^1^ compared to unvaccinated according to symptom group.

Symptom Category	Adjustments	Aged 12–14 Years				Aged 15–19 Years			
		N (n) ^2^	OR	95% CI	*p*	N (n) ^2^	OR	95% CI	*p*
**Cardiopulmonary symptoms**	**1. Unadjusted**	3679(188)	0.73	0.43–1.26	0.259	2590(114)	0.88	0.60–1.29	0.513
	**2. Adjusted ^3^**	3679(188)	0.74	0.43–1.27	0.270	2590(114)	1.00	0.67–1.48	0.989
	**3. Adjusted extended ^4^**	3666(188)	0.76	0.44–1.30	0.316	2563(112)	1.02	0.68–1.52	0.922
**CNS symptoms**	1	3855(202)	0.98	0.69–1.40	0.912	2656(119)	0.77	0.54–1.12	0.174
	2	3855(202)	0.94	0.65–1.34	0.728	2656(119)	0.78	0.53–1.14	0.201
	3	3844(202)	0.96	0.67–1.38	0.826	2630(117)	0.79	0.54–1.17	0.241
**Headaches**	1	3892(205)	0.97	0.73–1.29	0.834	2688(120)	1.00	0.68–1.47	0.992
	2	3892(205)	0.97	0.72–1.30	0.830	2688(120)	1.07	0.72–1.60	0.725
	3	3879(205)	0.98	0.73–1.31	0.900	2661(118)	1.08	0.72–1.61	0.721
**Gastrointestinal symptoms**	1	3851(200)	0.77	0.57–1.04	0.090	2668(119)	0.86	0.60–1.25	0.434
	2	3851(200)	0.78	0.58–1.06	0.119	2668(119)	0.91	0.62–1.34	0.625
	3	3839(200)	0.80	0.59–1.09	0.159	2640(117)	0.91	0.62–1.35	0.651
**Muscle or joint symptoms**	1	3404(175)	0.85	0.62–1.16	0.316	2316(107)	0.75	0.50–1.11	0.150
	2	3404(175)	0.85	0.62–1.16	0.306	2316(107)	0.79	0.53–1.18	0.253
	3	3393(175)	0.85	0.62–1.17	0.325	2290(105)	0.75	0.50–1.13	0.170
**Chills**	1	3854(201)	0.87	0.63–1.20	0.385	2671(121)	1.05	0.73–1.52	0.779
	2	3854(201)	0.86	0.62–1.19	0.366	2671(121)	1.11	0.77–1.61	0.574
	3	3841(201)	0.88	0.63–1.21	0.425	2643(119)	1.10	0.76–1.60	0.609
**Tiredness**	1	3907(205)	0.98	0.73–1.31	0.885	2719(121)	0.88	0.60–1.29	0.507
	2	3907(205)	0.95	0.70–1.27	0.709	2719(121)	0.94	0.63–1.40	0.761
	3	3894(205)	0.96	0.72–1.29	0.796	2691(119)	0.92	0.62–1.38	0.701

^1^ Symptoms reported 14 days after the first vaccine dose. Unvaccinated symptoms within 14 days before survey completion. ^2^ N = total number of participants included (after exclusion of participants with risk factors for outcome), n = number with reported symptoms. ^3^ Adjusted for: sex, age, along with register information on prevalent somatic disorders and psychiatric disorders. ^4^ Adjusted extend: In addition, adjusted for obesity, family income and period of survey response (for 15–19-year only). Participants with missing information are excluded.

**Table 4:** Odds ratio (OR) of short-term symptoms (first 14 days) after second dose vaccine against COVID-19: Results of logistic regression analyses on second dose vaccine population ^1^ compared to unvaccinated according to symptom group.

Symptom Category	Adjustments	Aged 12–14 Years				Aged 15–19 Years			
		N (n) ^2^	OR	95% CI	*p*	N (n) ^2^	OR	95% CI	*p*
**Cardiopulmonary symptoms**	**Unadjusted**	3540(49)	0.96	0.38–2.44	0.934	2817(341)	0.93	0.74–1.17	0.526
	**Adjusted ^3^**	3540(49)	0.90	0.35–2.30	0.825	2817(341)	0.74	0.58–0.94	0.015
	**Adjusted extended ^4^**	3527(49)	0.93	0.36–2.39	0.882	2789(338)	0.78	0.60–1.01	0.056
**CNS symptoms**	1	3703(50)	0.99	0.49–1.99	0.983	2898(361)	1.13	0.91–1.42	0.271
	2	3703(50)	0.89	0.44–1.81	0.757	2898(361)	1.03	0.81–1.31	0.836
	3	3692(50)	0.93	0.46–1.89	0.837	2872(359)	1.13	0.88–1.46	0.342
**Headaches**	1	3739(52)	2.28	1.30–4.00	0.004	2931(363)	1.85	1.43–2.40	0.000
	2	3739(52)	2.16	1.22–3.82	0.008	2931(363)	1.61	1.22–2.12	0.001
	3	3726(52)	2.20	1.24–3.90	0.007	2904(361)	1.66	1.24–2.22	0.001
**Gastrointestinal symptoms**	1	3703(52)	1.24	0.71–2.15	0.444	2911(362)	1.35	1.07–1.70	0.012
	2	3703(52)	1.19	0.68–2.08	0.536	2911(362)	1.18	0.91–1.51	0.211
	3	3691(52)	1.23	0.70–2.15	0.468	2882(359)	1.38	1.06–1.81	0.018
**Muscle or joint symptoms**	1	3275(46)	1.13	0.63–2.02	0.689	2513(304)	0.87	0.68–1.12	0.274
	2	3275(46)	1.14	0.63–2.04	0.669	2513(304)	0.76	0.59–0.99	0.043
	3	3264(46)	1.15	0.64–2.06	0.647	2487(302)	0.83	0.63–1.10	0.201
**Chills**	1	3705(52)	1.10	0.61–2.00	0.745	2916(366)	1.11	0.89–1.38	0.356
	2	3705(52)	1.11	0.61–2.01	0.734	2916(366)	0.97	0.77–1.23	0.808
	3	3692(52)	1.13	0.62–2.06	0.685	2887(363)	1.06	0.83–1.35	0.640
**Tiredness**	1	3754(52)	1.79	1.04–3.10	0.037	2967(369)	1.60	1.23–2.07	0.000
	2	3754(52)	1.65	0.95–2.87	0.077	2967(369)	1.35	1.02–1.78	0.035
	3	3741(52)	1.70	0.97–2.97	0.061	2938(366)	1.44	1.08–1.93	0.014

^1^ Symptoms reported 14 days after the first vaccine dose. Unvaccinated symptoms within 14 days before survey completion. ^2^ N = total number participants included (after exclusion of participants with risk-factors for outcome), n = number with reported symptoms. ^3^ Adjusted for: sex, age, along with register information on prevalent somatic disorders and psychiatric disorders. ^4^ Adjusted extend: In addition, adjusted for obesity, family income and period of survey response (for 15–19-year only). Participants with missing information are excluded.

**Table 5:** Odds ratio (OR) of long-term symptoms after COVID-19 vaccine in 15–19-year-olds: Results of logistic regression analyses on lasting at least two months ^1^ compared to unvaccinated according to symptom.

	Vaccinated N = 32	Non-Vaccinated N = 704	OR Unadjusted			OR adjusted ^2^		
			OR	95% CI	*p*	OR	95% CI	*p*
**Headache**	8 (25.0)	95 (13.5)	2.14	0.93–4.89	0.07	2.20	0.84–5.73	0.11
**Trouble remembering or concentrating**	9 (28.1)	136 (19.3)	1.63	0.74–3.61	0.22	1.01	0.39–2.63	0.98
**CNS symptoms**	6 (18.7)	109 (15.5)	1.26	0.51–3.13	0.62	0.80	0.27–2.40	0.69
**Cardiopulmonary symptoms**	5 (15.6)	83 (11.8)	1.39	0.52–3.70	0.51	0.47	0.14–1.58	0.22
**Gastrointestinal symptoms**	6 (18.7)	76 (10.8)	1.91	0.76–4.78	0.17	1.06	0.36–3.10	0.92
**Pain in muscles/joints**	n < 5	40 (5.7)	2.37	0.79–7.09	0.12	1.48	0.42–5.20	0.54
**Fatigue**	15 (46.9)	163 (23.2)	2.93	1.43–5.99	0.00	1.72	0.74–4.00	0.21
**Rashes**	n < 5	38 (5.4)	1.81	0.53–6.22	0.34	0.81	0.19–3.40	0.77
**Fever**	n < 5	n < 5	7.54	0.76–74.55	0.08	3.99	0.21–76.05	0.36
**Mood swings**	11 (34.4)	150 (21.3)	1.93	0.91–4.10	0.09	1.42	0.57–3.51	0.45

^1^ Symptoms reported 12–16 weeks after the second vaccine dose among vaccinated and prior to survey completion date among unvaccinated. ^2^ Adjusted for sex, age, obesity, binary measure of a certain prevalent somatic disease along with any prevalent psychiatric disease.

## Data Availability

Data cannot be made available for others due to privacy concerns and Danish data regulations.

## Supplementary Materials

The following supporting information can be downloaded at: https://www.mdpi.com/article/10.3390/vaccines10111863/s1. Supplementary Table S1: Grouping of short-term and long-term symptoms. Supplementary Table S2. Exclusion criteria according to symptom group: Prevalent health conditions as risk factors for outcome according to symptom group. Supplementary Table S3. Prevalent disorders assessed as confounders: Categories and definitions according to discharge diagnoses and filled prescription drugs. Supplementary Table S4. Use of prescription drugs. Supplementary Table S5. Proportion reporting categories of groups of symptoms during the last 14 days before survey completion: Participants vaccinated 14 days before survey completion versus unvaccinated. Supplementary Table S6. Characteristics of vaccinated and unvaccinated adolescent survey responders versus non-responders.
