# Supplementary material for: Short- and Long-Term Self-Reported Symptoms in Adolescents Aged 12–19 Years after Vaccination against SARS-CoV-2 Compared to Adolescents Not Vaccinated—A Danish Retrospective Cohort Study
Source: Vaccines (Basel). 2022 Nov 4;10(11):1863. doi: 10.3390/vaccines10111863 (PMC9692356; doi:10.3390/vaccines10111863)
Supplement: Supplementary file 1 [file vaccines-10-01863-s001.zip › vaccines-1959739-supplementary.pdf]

Contents supplementary

Supplementary Table S1. Grouping of short-term and long-term symptoms.

**Short-term symptoms**	
**Symptom group**	**Symptoms**
CNS symptoms	Numbness or tingling in parts of your body<br>Blurred vision<br>Heavy feelings in your arms or legs (when they feel too heavy to move)<br>Faintness or dizziness
Cardiopulmonary symptoms	Your heart beating too fast (even when you're not exercising)<br>Trouble getting your breath (when you're not exercising)<br>Pain in your heart or chest
Gastrointestinal symptoms	Nausea or upset stomach<br>Vomiting<br>Loose (runny) BMs or diarrhea<br>Pain in your stomach or abdomen
Muscle or joint symptoms	Pains in your lower back<br>Sore muscles<br>Pain in your knees. elbows or other joints<br>Pain in your arms or legs
Headache	Headache
Chills	Hot or cold spells (suddenly feeling hot or cold for no reason)
Tiredness	Weakness (feeling weak) in parts of your body<br>Feeling low in energy or slowed down
**Long-term symptoms**	
Headache	
Trouble remembering or concentrating	
CNS symptoms	Dizziness when standing<br>Dizziness
Cardiopulmonary symptoms	Trouble breathing<br>Chest pain<br>Palpitations
Gastrointestinal symptoms	Stomach aches<br>Nausea
Pain in muscles/joints	
Fatigue	
Rashes	
Fever	
Mood swings	

Supplementary Table S2. Exclusion criteria according to symptom group: Prevalent health conditions as risk factors for outcome according to symptom group.

Survey symptom group	Prevalent health condition[1]: Prior diagnosis (ICD) and Filled prescription drug (ATC)	Look back period[2]
**Muscle and joint conditions** (Pains in your lower back. Sore muscles. Pain in your knees. elbows or other joints. Pain in your arms or legs)	**ICD:**	
	Infectious/non-chronic conditions: G61; M00-M03; M65-M79	Medium
	Rheumatological disorders: M13-M14;	Short
	Rheumatological disorders: M46; M49	Medium
	Rheumatological disorders: M05-M09; M45; M47-M48; M31-34	Full
	Muscle and joint disorders: G70-G71; M20-M25; M60-M79	Full
	Congenital Musculoskeletal deformation: Q65-Q74; Q76-Q79 M40-M54	Full
	Injuries: S20-S99	Short
	**ATC**:	
	Musculoskeletal/ Analgetics: M01; M02; N02A; N02B	Short
**CNS conditions** (Numbness or tingling in parts of your body. Blurred vision. Heavy feelings in your arms or legs (when they feel too heavy to move). Faintness or dizziness)	**ICD**:	
	Episodic /paroxysmal disorders: G40; G41	Full
	Nervous system disorders: G82; G94; G96	Full
	Congenital nervous system malformation: Q05-Q07	Full
	Seizures. non-epileptic: R53; R56	Medium
	Symptoms signs Nervous System: R25	Medium
	Inflammatory diseases of nervous system: G00-G09 A80-89	Medium
	Cerebral palsy-paralytic syndrome/congenital: G80-G83; Q00-Q04	Full
	Cerebrovascular diseases. blurred vision: I60-I69; H53	Full
	**ATC**:	
	Antiepileptics; N03A	Medium
	Spasmolytics: M03AX; M03B	Medium
**Headache** (Headache)	**ICD10**	
	Migraine/headaches: G43- G44; N943; R51	Medium
	Injuries head/neck: S00-S19	Short
	**ATC**	
	N02C	Medium
**Cardiovascular conditions** (Your heart beating too fast (even when you're not exercising). Pain in your heart or chest)	**ICD**:	
	Ischemic & Pulmonary heart diseases: I20-I28	Full
	Chronic heart disease: I00-09; I31; I34-39; I41-43; I50-52	Full
	Infectious heart disease: A395; I30-33; I40	Medium
	Arrythmias. conduct-disorders/tachycardias: I44-I49	Full
	Congenital heart disease: Q20-Q28	Full

	**ATC**	
**Respiratory conditions**<br>(Trouble getting your breath (when you're not exercising))	**ICD**	
	Infectious resp diseases: J00-22; J40; B34 (excl. 34.2)	Very short
	Chronic lower resp diseases; ischemic/pulmonary heart diseases: J40-47; I20-28;	Full
	Chronic heart disease: I00-I09; I31; I34-39; I41-43; I50-52	Full
	Infectious heart disease: A395; I30- I33; I40	Medium
	Injuries. pneumothorax: S20-26; J93	Short
	Congenital resp/heart diseases: Q30-34; E84; Q20-Q28	Full
	**ATC**	
	Short acting agents (SAB): R03AC2- R03AC4; R03AL1-2; R03CC02	Short
	Bronchodilating comb: R03AC12- R03AC19; R03BB01; R03BB4- R03BB7; R03AL11-12	Medium
	Agents comb with Corticosteroids: R03BA; R03AK; R03AL08; R03AL09	Medium
	Leukotriene receptor antagonist: R03DC03	Medium
**Gastrointestinal conditions**<br>(Nausea or upset stomach.<br>Vomiting.<br>Loose (runny) BM's or diarrhea.<br>Pain in your stomach or abdomen)	**ICD**:	
	Infectious & non-chronic GI conditions: K20. K25-29. K35-K38; K60-K67; A00-A09; K90-K93	Short
	Autoimmune GI: K50-K52; except K52.2	Full
	Functional GI: K21-K23; K30-K31; K56-K59	Medium
	Congenital GI: Q38-Q45	Full
	Toxic GI: K70-K77; K80-K87	Full
	**ATC**:	
	Agents in acid disorders. constipation. diarrhea. nausea: A02; A04; A07E	Short
	Agents in functional/autoimmune GI disorders: A03	Medium
**Febrile conditions**<br>(Hot or cold spells (suddenly feeling hot or cold for no reason))	**ICD**	
	Infectious diseases: A00-09; A15-19; A20-28; A30-49; A50-89; A92-99; B00.2-7; B01-02 excl. B01.9. B02.3. B02.9; B05-06; B08-09 excl. B08.1; B15-17; B19-20; B22-34 (excl. B22.2; B30; B34.2); B37.5-7; B38-49 (excl. B38.3. B40.3. B43.0. B45.2. B46.3); B50-54; B55.0; B56-64 (excl. B57.2-5. B58.0); B65-67	Very short
	Organ specific infectious diseases: G00-09; H66.0; H70.0; I00-01; I30-33; I38; I40; J00-22; J40; J85-86; K35; K61; M00-03; M72.6; N10; N12; N13.6; N61	Very short
	Inflammatory diseases: M05-14. M30-36; M60.0; M70.0; N70.0; N71.0	Long
	Miscellaneous: E85.0A	Long
	Drug induced fever: R50.2	Very short
	**ATC**	
	Systemic antibiotics: J01	Very short
**No applied risk-factor**<br>(Weakness (feeling weak) in parts of your body.		

Feeling low in energy or slowed down)		

[1] Seven groups of risk factors for outcome were assessed corresponding to ICD10 primary discharge diagnoses and/or prescription drug fillings (ATC) at a certain look-back prior to survey completion.

[2] Looking back periods from the survey completion date: Full (ICD: full historic); Medium (ICD: 1 year; ATC: 6 months); Short (ICD: 3 months; ATC: 2 month); Very short: 14 days (ICD & ATC).

Supplementary Table S3. Prevalent disorders assessed as confounders: Categories and definitions according to discharge diagnoses and filled prescription drugs.

**Disorder categories**	**Register-based disorder defintions[1]: Prior diagnosis (ICD 10) / filled of prescription drug (ATC)**	**Look back period[2]**
Asthma[3]	**ICD10**	
	J45-J46	Full
	**ATC**	
	Bronchodilating agents(comb): R03BA; R03AK; R03AL08; R03AL09; R03AL11-12; R03AC12-R03AC19; R03BB01;	Medium
	Leukotriene receptor antagonist: R03DC03	Medium
Other respiratory disorders[3]	**ICD10**	
	E84; J41-44; J47; J84; J96.1; P27;	Full
	J40	Short
	**ATC**	
	-	
Cardiovascular disorders[3]	**ICD10**	
	I05-I09; I27; I34-39; I41-I47; I50-I52	Full
	I01; I31; I33; I40	Medium
	**ATC**	
	-	Medium
Renal disorders incl. dialysis[3]	**ICD10:**	
	N03; N04-5; N07-8; N18-19; N25-N27; Y84.1	Full
	N00-1; N17	Medium
	**ATC:**	
	-	
Diabetes mellitus I or II[3]	**ICD10:**	
	E10-E11; Z96.4	Full
	**ATC:**	
	A10A	Medium
Endocrine disorders. excl. diabetes[3]	**ICD10**	
	E03.3; E03.5-9; E05-06; E27.1; E27.3-4	Full
	E27.2	Short
	**ATC**	
	-	
Hematologic disorders[3]	**ICD10**	

	D59.0-1; D69.0; D69.3; D86	Full
	**ATC**	
	-	Medium
Dermatologic diseases[3]	**ICD10**	
	L10; L12; L13.0	Medium
	L20; L40; L63; L80	Full
	**ATC**	
Rheumatological disorders[3]	**ICD10**	
	M05-M09; M30-36; M45; M60 (excl. M60.0)	Full
	**ATC**	
	-	
Neuromuscular disorders[3]	**ICD10**	
	G35-37; G70-71; G80; G82	Full
	G61;	Medium
	**ATC**	
	-	Medium
Gastrointestinal disorders incl. cirrhosis (any cause) [3]	**ICD10**	
	K50-51; K70.2-3; K71.7; K74; K76.1; K78.8; K900;	Full
	K35	Short
	**ATC**	
		Medium
Congenital malformations and chromosomal abnormalities[3]	**ICD10**	
	Q20-28; Q31-34; Q90-95	Full
Malignancy[3]	**ICD10**	
	C00-97	Full
	**ATC**	
	L01	Full
Organ transplantation or immunodeficiency[3]	**ICD 10**	
	Organ transplantation: Z94 excl. Z94.5. Z94.7	Full
	Immunodeficiency: D70-72; D730; D80-84	Full
	**ATC**	
	L04	Full
Psychiatric disorders[4]	ICD10	
	F (any psychiatric primary diagnoses)	Full

[1] Individuals with a list disorders within a look back period prior to survey completion date were identified, applying individual-level register information, corresponding to primary discharge diagnoses (ICD-10 codes) and/or prescription drug use disease proxies (ATC codes). The list includes somatic health conditions in adolescents conferring an increased risk of SARS-CoV-2 infection[21] along with any psychiatric discharge disorders.

[2] Looking back periods from the survey completion date:
Full (ICD: full historic); Medium (ICD: 1 year; ATC: 6 months); Short (ICD: 3 months; ATC: 2 month); Very short: 14 days (ICD & ATC).
[3] Included as a joint binary measure for somatic disease confounder: Yes (Yes in any of the included prevalent disease) No (No as to all included prevalent disorders).
[4] Included as a specific psychiatric disease confounder.

Supplementary Table S4. Use of prescription drugs.

Filled prescription drug prior to survey completion		Look back period[1]
**Drug category**	**Anatomic Therapeutic Chemical (ATC) Classification**	
Short-acting bronchodilating (BL) acting agents	R03AC02- 04; R03AL01-02; R03CC02	Short
Long-acting BL agents incl. Leukotriene receptor antagonists	R03AC02-04; R03AC12-19; R03AK; R03AL01-09; R03AL11-12; R03BA; R03BB01; R03BB04-07; R03DC03	Medium
Systemic antibiotic	Systemic antibiotics: J01	Very Short
Paracetamol	N02BE01; N02BE51	Medium
NSAID; incl. Acetylsalicylic Acid	M01A (excl. M01AX); N02AJ07; N02BA01	Medium

[1] Looking back periods from the survey completion date: Medium (ATC: 6 months); Short (ATC: 2 month); Very short: (ATC 14 days).

Supplementary Table S5. Proportion reporting categories of groups of symptoms during the last 14 days before survey completion: Participants vaccinated 14 days before survey completion versus unvaccinated.

Group of survey symptoms	Age-group (years)	14 days after 1. vaccine						14 days after 2. vaccine					
		Total	Unvaccinated		Vaccinated			Total	Unvaccinated		Vaccinated		
			N	Symptom (%)	N	Symptom (%)	p		N	Symptom (%)	N	Symptom (%)	p
CNS symptoms[1]	**12-14**	3855	3653	20.1	202	19.8	0.37	3703	3653	20.1	50	20.0	0.72
	**15-19**	2656	2537	55.9	119	49.6		2898	2537	55.9	361	59.0	
Cardiopulmonary symptoms[2]	**12-14**	3898	3694	11.0	204	9.3	0.96	3746	3694	11.0	52	9.6	0.84
	**15-19**	2709	2589	44.2	120	40.0		2955	2589	44.2	366	42.9	
Gastrointestinal symptoms[3]	**12-14**	3851	3651	39.0	200	33.0	0.64	3703	3651	39.0	52	44.2	0.78
	**15-19**	2668	2549	59.9	119	56.3		2911	2549	59.9	362	66.9	
Muscle or joint symptoms[4]	**12-14**	3404	3229	42.7	175	38.9	0.61	3275	3229	42.7	46	45.7	0.43
	**15-19**	2316	2209	65.7	107	58.9		2513	2209	65.7	304	62.5	
Headache[5]	**12-14**	3892	3687	41.2	205	40.5	0.89	3739	3687	41.2	52	61.5	0.51
	**15-19**	2688	2568	64.9	120	65.0		2931	2568	64.9	363	77.4	
Chills[6]	**12-14**	3854	3653	28.7	201	25.9	0.43	3705	3653	28.7	52	30.8	0.99
	**15-19**	2671	2550	44.2	121	45.5		2916	2550	44.2	366	46.7	
Tiredness[7]	**12-14**	3907	3702	37.6	205	37.1	0.66	3754	3702	37.6	52	51.9	0.71
	**15-19**	2719	2598	69.0	121	66.1		2967	2598	69.0	369	78.1	

Supplementary Table S6. Characteristics of vaccinated and unvaccinated adolescent survey responders versus non-responders.

	**Non-responders**			**Responders**		
	**All**	**Vaccinated**	**Unvaccinated**	**Vaccinated first dose**	**Vaccinated second dose**	**Unvaccinated**
	(n=142,715)	(n=86,694)	(n=56,021)	(n=326)	(n=421)	(n=6,300)
Age group 12-14 years, n (%)	46,313 (32·5%)	20,442 (23·6%)	25,871 (46·2%)	205 (62·9%)	52 (12·4%)	3702 (58·8%)
Age group 15-19 years, n (%)	96,402 (67·5%)	66,252 (76·4%)	30,150 (53·8%)	121 (37·1%)	369 (87·6%)	2598 (41·2%)
Sex (Girl), n (%)	67,284 (47·1%)	40,932 (47·2%)	26,352 (47·0%)	167 (51·2%)	251 (59·6%)	3210 (51·0%)
**Risk factors for outcome, n (%)**[1]						
Muscle and joint conditions	21,167 (14·8%)	13,557 (15·6%)	7610 (13·6%)	44 (13·5%)	71 (16·9%)	862 (13·7%)
Cardiovascular conditions	545 (0·4%)	354 (0·4%)	191 (0·3%)	(n<5)	(n<5)	17 (0·3%)
CNS conditions	2918 (2·0%)	1781 (2·1%)	1137 (2·0%)	5 (1·5%)	10 (2·4%)	110 (1·7%)
Headache	1390 (1·0%)	908 (1·0%)	482 (0·9%)	(n<5)	6 (1·4%)	45 (0·7%)
Gastrointestinal conditions	2443 (1·7%)	1521 (1·8%)	922 (1·6%)	7 (2·1%)	7 (1·7%)	100 (1·6%)
Respiratory conditions	7916 (5·5%)	4851 (5·6%)	3065 (5·5%)	24 (7·4%)	31 (7·4%)	333 (5·3%)
Febrile conditions	2584 (1·8%)	1667 (1·9%)	917 (1·6%)	(n<5)	(n<5)	97 (1·5%)
**Prevalent disorders as confounders, n (%)**[2]						
Asthma and other respiratory disorders	6504 (4·6%)	3998 (4·6%)	2506 (4·5%)	20 (6·1%)	24 (5·7%)	290 (4·6%)
Cardiovascular or renal disorders	601 (0·4%)	371 (0·4%)	230 (0·4%)	(n<5)	(n<5)	22 (0·3%)
Gastrointestinal disorders	691 (0·5%)	468 (0·5%)	223 (0·4%)	(n<5)	(n<5)	43 (0·7%)
Endocrine disorders including diabetes	995 (0·7%)	685 (0·8%)	310 (0·6%)	(n<5)	7 (1·7%)	33 (0·5%)
Haematological disorders	571 (0·4%)	339 (0·4%)	232 (0·4%)	-	(n<5)	39 (0·6%)
Rheumatological disorders	451 (0·3%)	242 (0·3%)	209 (0·4%)	-	(n<5)	23 (0·4%)
Neuromuscular disorders	500 (0·4%)	302 (0·3%)	198 (0·4%)	(n<5)	(n<5)	11 (0·2%)
Congenital malformations & chromosomal abnormalities	1077 (0·8%)	637 (0·7%)	440 (0·8%)	(n<5)	5 (1·2%)	39 (0·6%)
Malignancy or organ transplantation	537 (0·4%)	326 (0·4%)	211 (0·4%)	(n<5)	(n<5)	31 (0·5%)
Any of the above somatic disorders	18,474 (12·9%)	11,628 (13·4%)	6846 (12·2%)	49 (15·0%)	71 (16·9%)	763 (12·1%)
Psychiatric disorder	11,667 (8·2%)	7312 (8·4%)	4355 (7·8%)	25 (7·7%)	37 (8·8%)	518 (8·2%)
**Prevalent prescription-drug use, n (%)**						

Short- acting bronchodilating agents.	1068 (0·7%)	660 (0·8%)	408 (0·7%)	(n<5)	(n<5)	39 (0·6%)
Long-acting bronchodilating agents.	3026 (2·1%)	2017 (2·3%)	1009 (1·8%)	10 (3·1%)	9 (2·1%)	136 (2·2%)
Systemic antibiotics	1267 (0·9%)	899 (1·0%)	368 (0·7%)	(n<5)	(n<5)	35 (0·6%)
Analgesics: Paracetamol	2460 (1·7%)	1684 (1·9%)	776 (1·4%)	(n<5)	12 (2·9%)	86 (1·4%)
Analgesics: NSAIDs	3194 (2·2%)	2247 (2·6%)	947 (1·7%)	7 (2·1%)	13 (3·1%)	125 (2·0%)
**Parental socio-economic position, n (%)**						
Highest attained parental education						
Basic education	11,478 (8·0%)	4167 (4·8%)	7311 (13·1%)	9 (2·8%)	11 (2·6%)	296 (4·7%)
High school and vocational training	60,929 (42·7%)	36,637 (42·3%)	24,292 (43·4%)	112 (34·4%)	182 (43·2%)	2382 (37·8%)
Higher education	68,615 (48·1%)	45,433 (52·4%)	23,182 (41·4%)	204 (62·6%)	227 (53·9%)	3598 (57·1%)
Missing	1693 (1·2%)	457 (0·5%)	1236 (2·2%)	(n<5)	(n<5)	24 (0·4%)
Annual family income						
Low (1. tertile)	48,688 (34·1%)	22,986 (26·5%)	25,702 (45·9%)	71 (21·8%)	95 (22·6%)	2047 (32·5%)
Middle (2. tertile)	41,959 (29·4%)	26,338 (30·4%)	15,621 (27·9%)	108 (33·1%)	127 (30·2%)	2025 (32·1%)
High (3. Tertile)	50,056 (35·1%)	36,586 (42·2%)	13,470 (24·0%)	145 (44·5%)	196 (46·6%)	2189 (34·7%)
Missing	2012 (1·4%)	784 (0·9%)	1228 (2·2%)	(n<5)	(n<5)	39 (0·6%)

[1] Risk-factors for outcome (i.e., reported symptoms) correspond to registered prior hospital diagnoses (and medicine proxies) implying groups of reported symptoms (as defined in Supplementary Table S2).

[2] Prevalent disorders included as confounders in the analyses: The included somatic disorders are included as one somatic disorder binary variable (1 if any, 0 if none). Psychiatric disorder if any registered F- ICD code.
